# Renal protection in cardiovascular surgery

**DOI:** 10.12688/f1000research.7348.1

**Published:** 2016-03-11

**Authors:** Nora Di Tomasso, Fabrizio Monaco, Giovanni Landoni

**Affiliations:** 1 Department of Anesthesia and Intensive Care, IRCCS San Raffaele Scientific Institute, Milan, Italy; 2 Vita-Salute San Raffaele University, Milan, Italy

**Keywords:** Acute kidney injury, cardiovascular surgery, biomarkers

## Abstract

Acute kidney injury (AKI) is one of the most relevant complications after major surgery and is a predictor of mortality. In Western countries, patients at risk of developing AKI are mainly those undergoing cardiovascular surgical procedures. In this category of patients, AKI depends on a multifactorial etiology, including low ejection fraction, use of contrast media, hemodynamic instability, cardiopulmonary bypass, and bleeding. Despite a growing body of literature, the treatment of renal failure remains mainly supportive (e.g. hemodynamic stability, fluid management, and avoidance of further damage); therefore, the management of patients at risk of AKI should aim at prevention of renal damage. Thus, the present narrative review analyzes the pathophysiology underlying AKI (specifically in high-risk patients), the preoperative risk factors that predispose to renal damage, early biomarkers related to AKI, and the strategies employed for perioperative renal protection. The most recent scientific evidence has been considered, and whenever conflicting data were encountered possible suggestions are provided.

## Introduction/Pathogenesis

Renal damage is the first step of a potentially fatal cascade leading to kidney failure ^1^. Therefore, it is considered an independent risk factor for death in the general population undergoing surgery ^2^. Timely detection of early signs of renal damage, together with preventive kidney measures and renal protection, plays a key role in the patient’s outcome ^3^, potentially reducing mortality, hospital length of stay, and costs ^4, 5^.

Acute kidney injury (AKI) is defined as loss of excretory function, accumulation of nitrogen side-products, and reduction in urinary output, all leading to volume overload ^6^. This corresponds, within 48 hours, to an increase in serum creatinine (sCr) between 1.5 to 1.9 times the baseline sCr (AKI stage 1), 2 to 2.9 times baseline (AKI stage 2), or more than 3 times baseline (AKI stage 3) and respectively to a reduction in urinary output of less than 0.5 mL/kg per hour for 6 to 12 hours, of less than 0.5 mL/kg per hour for more than 12 hours, and of less than 0.3 mL/kg per hour for 24 hours or anuria ^7, 8^.

The pathogenesis behind AKI is extremely complex and dependent on a variety of factors; however, in the perioperative setting, one of the most common causes is prolonged hypoperfusion (pre-renal AKI) associated with septic shock, cardiogenic shock, hypovolemia, and bleeding. Moreover, perioperative use of nephrotoxic drugs and contrast medium may further impair renal function ^8^. Therefore, key steps in improving renal function and outcome are the identification of patients at high risk of AKI, avoidance of nephrotoxic agents, and adoption of protective renal strategies.

With the present review, we aim at analyzing, specifically in the cardiovascular surgery setting, possible risk factors for kidney damage, perioperative strategies for renal protection and optimization, and identifying potential screening tests able to determine patients at higher risk of AKI.

## Patients’ stratification

Patients’ stratification to detect those at high risk of AKI is the first step to guarantee an individualized management ^7^. The vast majority of randomized control studies and meta-analyses on perioperative renal protection measures were performed on patients undergoing cardiovascular surgeries. These patients are at increased risk of renal damage and failure due to preoperative critical heart function, the need for diagnostic procedures requiring contrast media, the effect of cardiopulmonary bypass (CPB), and the increased incidence of bleeding and low cardiac output syndrome (LCOS) ^9^. More specifically, chronic kidney disease (CKD), systolic or diastolic left ventricular dysfunction, diabetes, and drugs such as angiotensin-converting enzyme inhibitors, angiotensin receptor blockers, non-steroidal anti-inflammatory drugs (NSAIDs), and intravenous contrast medium are all preoperative factors that increase the risk of developing postoperative AKI ^10^. On the other hand, complex cardiac surgery procedures with prolonged CPB and aortic cross-clamp time, hemodilution, and protracted low blood pressure are all intraoperative conditions that may further damage renal function.

Several predictive scores for cardiac surgery-related AKI have been proposed as prognostic elements for anticipating patient treatment. The AKICS ^11^ (Acute Kidney Injury following Cardiac Surgery) score, for example, evaluates preoperative (age of more than 65, preoperative creatinine of more than 1.2 mg/dL, preoperative capillary glucose of more than 140 mg/dL, and heart failure), intraoperative (combined surgeries and CPB time of more than 2 hours), and postoperative (low cardiac output [CO] and low central venous pressure) parameters associated with AKI. It seems to accurately predict post-cardiac surgery AKI.

The score by Thakar *et al.* ^12^, which considers the major preoperative risk factors, such as higher sCr, diabetes, chronic obstructive pulmonary disease, previous cardiac surgery, severe cardiovascular disease, and female gender, seems to have the highest predictive value ^13^ in the discrimination of patients at risk of developing Kidney Disease Improving Global Outcomes AKI (KDIGO-AKI).

Another interesting score for predicting the risk of postoperative dialysis, developed from a huge dataset of 449,524 patients by Mehta *et al.* ^14^, showed that the risk of postoperative dialysis is correlated with preoperative kidney function (sCr of at least 2.6 mg/dL indicates extreme risk for dialysis), but other important factors can also influence postoperative renal function, such as advanced age and insulin-dependent diabetes, which are associated with glomerular sclerosis, and chronic respiratory disease, associated with prolonged ventilation.

Although these scores provide a good starting point for early management of patients at risk for AKI, unfortunately no established and well-performing scoring system in cardiac surgery is able to stratify patients according to their risk of developing AKI. Great debate and further investigations are needed on the use of available mortality scores, like the additive EuroSCORE ^15^.

## Diagnostic tests and biomarkers

sCr and urinary output are routinely performed to assess renal function—Acute Kidney Injury Network (AKIN) and Risk, Injury, Failure, Loss of kidney function, and End-stage kidney disease (RIFLE) scores—even if both of them can be considered late (sCr increases only when glomerular filtration decreases by more than 50%) and indirect expressions of kidney damage ^6^. More recent studies on functional genomics and proteomics have identified possible renal biomarkers that are still under investigation and that may act as early markers of renal impairment. Among these, the most promising are neutrophil gelatinase-associated lipocalin (NGAL), a “real-time” serum and urinary marker of tubular stress/injury ^16^, and cystatin C (CyC), which detects changes in glomerular filtration rates ^17– 19^. NGAL has also been evaluated as a sensible marker of renal recovery in critically ill patients ^20, 21^, and CyC has shown a high rate of false-positive results in acute inflammation states (for example, following CPB) ^22^. However, in complex cases with multiple comorbidities, the combination of CyC and NGAL may increase the sensitivity and specificity of such tests ^23^. Recently, Prowle *et al.* ^24^ have shown a possible correlation between serum and urinary levels of hepcidin in the 24 hours following cardiac surgery and postoperative AKI. However, these results require further investigation.

Moreover, in different clinical settings such as severe sepsis and septic shock, markers such as liver-type fatty acid-binding protein (L-FABP) ^25^, interleukin-18 (IL-18), kidney injury molecule-1 (KIM-1) ^26^, and endogenous ouabain ^27^, an adrenal gland stress hormone, have shown a good discriminant power between patients who will develop AKI and those who will not ^23^. Although preliminary results seem promising and useful in clarifying the pathophysiology of renal dysfunction, new renal biomarkers are still not widely adopted in clinical practice and require further investigation.

## Contrast-induced acute kidney injury

No specific treatment has been identified as effective in preventing AKI, and only few measures have been shown to reduce mortality in such patients ^6^. Avoidance of nephrotoxic drugs, like NSAIDs, aminoglycosides, and radio-contrast agents, can significantly reduce the risk of AKI occurrence ^7^. Among these, the use of contrast media is relevant, as it is associated with the so-called contrast-induced AKI (CI-AKI) ^28^. Contrast medium affects kidney perfusion, mainly involving the renal medulla, causing a modest and transient decrease in renal blood flow. This leads to ischemia and tubular cell injury, which accounts for a considerable percentage of in-hospital cases of renal dysfunction ^29^. CI-AKI is commonly defined as an increase of 25% or more (>0.5 mg/dL) in sCr levels 48 hours following contrast medium injection ^30^. Specific results regarding CI-AKI in cardiac surgery are still lacking, and great debate persists. Hennessy *et al.* observed AKI onset after contrast agent administration, especially in patients undergoing valve surgery ^31^. On the contrary, several other authors have not reported a worsening of renal function in the overall population when contrast media was administered the day before the surgical procedure ^32– 34^. Interestingly, Calzavacca *et al.* ^35^, in a randomized cross-over experimental study, noted that administration of radio-contrast media does not induce vasoconstriction injury or a reduction of renal blood flow. A possible explanation for this finding may be that low-risk patients, with an overall uneventful perioperative course and preserved renal function, maintain adequate/sufficient kidney flow reserve, resulting in subclinical organ damage. On the other hand, high-risk patients, with CKD ^31, 36^, previous nephrectomy, or hemodynamic instability, further lose the capacity of renal blood flow auto-regulation when contrast medium is administered, resulting in medullary ischemia ^37^. Moreover, in this specific population subset, AKI most commonly results from the overlap of baseline clinical characteristics, CI-AKI, and cardiovascular surgery-related injury ^38^.

Because AKI is such a prominent in-hospital complication, many studies have been conducted to try to identify possible predictors of CI-AKI. Mehran *et al.* ^30^, after analyzing 8357 patients undergoing percutaneous coronary intervention (PCI), proposed a clinical score including the patient’s demographics (age and sex), medical history (CKD: sCr >1.5 mg/dL), hematocrit (Ht), diabetes, advanced NYHA (New York Heart Association) class, previous pulmonary edema, and procedure-related variables (systolic arterial blood pressure of less than 80 mmHg and need for inotropic drugs or mechanical circulatory support or both) to predict the onset of post-procedural CI-AKI. Further considerations involve the administered volume of contrast medium, an elevated contrast medium volume-to-glomerular filtration rate ratio, and low left ventricular ejection fraction. Therefore, according to the Mehran Score, patients scoring positive for at least three risk factors will have a 26% probability of developing CI-AKI and a 1% probability of needing dialysis following PCI ^30^.

Strategies or drugs capable of preventing and reducing the risk of CI-AKI are still few and largely debated ^39^. In clinical practice, perioperative hydration is commonly used along with an intravenous isotonic saline infusion rate of 1 mL/kg per hour from 4 to 12 hours before the procedure to 18 to 24 hours after the procedure ^40^. Particular attention should be paid when dealing with heart failure patients for an increased risk of volume overload and exacerbation of unstable hemodynamic condition. Other measures are reduced volume of contrast medium, preferably using iso-osmolar or low-osmolar iodinated contrast medium ( Table 1), and urine alkalinization with sodium bicarbonate despite conflicting results ^41^. Several prospective studies and meta-analyses have analyzed N-acetylcysteine (NAC), reporting protective effects on renal function due to antioxidant properties ^42^. However, the lack of well-designed randomized control studies with adequate sample size and statistical power and subsequent trials with negative findings strongly limited the widespread use of the compound ^43– 45^. Most of the previously reported limitations may be overcome with the conclusion of the ongoing large, adequately powered, randomized controlled trial (RCT) on the prevention of serious adverse events following angiography (PRESERVE). This study was designed to test the efficacy of sodium bicarbonate and NAC in the prevention of CI-AKI following angiography ^43^. In this context, fenoldopam has shown no benefits in patients with CKD ^45^.

**Table 1.** Perioperative renal protection drugs, techniques, and strategies to be avoided in cardiovascular surgery.

Primum non nocere*	Standard patients	Patients at high risk of AKI
Nephrotoxic drugs (aminoglycosides, NSAIDs, ACEIs, and cyclosporine)	Avoid routine use of nephrotoxic drugs in your department: - Use different antibiotics in your protocols - Use other painkillers - Stop ACEIs at hospital admission or use short-lived ones - Consult specialist for other, less nephrotoxic, drugs	Avoid nephrotoxic drugs in these patients If you consider them lifesaving in a specific patient, use strict monitoring of blood concentration (when possible) and renal function Consider postponing surgery
Contrast medium	Avoid routine use of high-volume dose and hyperosmolar iodinated contrast medium Avoid routine use of contrast medium the day of surgery	Avoid any unnecessary imaging Avoid high-volume dose and hyperosmolar iodinated contrast medium Avoid administration less than 24 hours before surgery Consider postponing surgery by several days even if there was no serum creatinine increase after the contrast medium procedure
Anemia	Avoid routine Ht of less than 21%	Avoid Ht of less than 24%
Hypotension	Avoid long periods of hypotension as routine in your center (start of CPB, control of bleeding) At the same time, avoid routine use of inotropes and vasoconstrictors	Avoid MAP of less than 65 mmHg during CPB Use fluids, transfusions, inotropes, and vasoconstrictors to reach your targets
Fluid management	Avoid fluid overload and routine use of HES	Avoid fluid overload and HES Avoid hypovolemia
Diuretics	Avoid routinely using diuretics to prevent and treat AKI	Avoid using diuretics to prevent and treat AKI In the management of volume overload, consider using continuous infusion instead of boluses
Dopamine	Avoid routine use of low-dose dopamine to prevent AKI	Avoid using low-dose dopamine to prevent or treat AKI
Aspirin	Avoid routine use of aspirin to prevent AKI in the perioperative period	Avoid using NSAIDs

*First do not harm. ACEI, angiotensin-converting enzyme inhibitor; AKI, acute kidney injury; CPB, cardiopulmonary bypass; HES, hydroxyethyl starch; Ht, hematocrit; MAP, mean arterial pressure; NSAID, non-steroidal anti-inflammatory drug.

To date, even current guidelines are divided between KDIGO, which recommends volume expansion and oral NAC only in patients at risk of CI-AKI, and the American College of Cardiology, American Heart Association, and Society for Cardiovascular Angiography, which discourage instead NAC administration.

## Acute kidney injury in cardiac surgery

The incidence of AKI following cardiac surgery varies from 0.3% to 30%, and 3% of patients require temporary or long-term renal replacement therapy (RRT). Ischemia-reperfusion injury, exogenous and endogenous toxins, metabolic factors, oxidative stress, micro-embolization, neuro-humoral activation, inflammation, non-pulsatile CPB flow, and hemodynamic instability are the main determinants of AKI ^46^. These factors can be present at the same time, acting synergistically or more often occurring randomly and with different importance.

CPB is strongly believed to play a fundamental role in the development of AKI; thus, whenever possible, CPB avoidance is desirable. A recent, large RCT comparing patients undergoing coronary bypass surgery with or without CPB showed a reduced risk of postoperative AKI after off-pump coronary artery bypass grafting (CABG) surgery, although it failed to demonstrate a better preserved kidney function at 1 year ^47^. Therefore, hemodynamic optimization rather than CPB utilization is the cornerstone for AKI prevention.

Consequently, perioperative hypotension should be avoided and a mean arterial pressure (MAP) able to guarantee an adequate glomerular capillary filtration pressure is desirable. Although there is no optimal target MAP demonstrated to reduce the risk of AKI during CPB ^48^, MAP values of more than 60 to 65 mmHg are a reasonable threshold to secure adequate renal perfusion. In patients with a clinical history reporting chronic hypertension or diabetes, higher MAP values should be considered.

According to physiology, the kidneys are the second organ in terms of oxygen consumption and receive a high amount of blood flow. Nevertheless, the oxygen extraction fraction is low compared with other organs, making the kidneys extremely sensitive to changes in blood flow. Acute alterations in oxygen delivery (DO _2_) occurring during CPB may be responsible for renal function impairment. In fact, DO _2_ is given by the product between Ht and CO, the latter being replaced by pump flow during CPB; therefore, greater hemodilution corresponds to a lower DO _2_, unless CO or pump flow is increased. In a recent single-center retrospective cohort study, Ranucci *et al.* explored the relationship between low Ht levels (Ht of less than 24%) during CPB, decreased renal oxygen delivery, and postoperative AKI ^49^. They found that strategies aiming at reducing hemodilution during CPB are effective in reducing AKI. This further recommends maintaining Ht levels above such a threshold and concomitantly setting the CO/pump flow according to the level of Ht. In the perioperative setting of cardiac and non-cardiac surgery, a liberal transfusion strategy has been recently proposed ^50^ as not detrimental and even beneficial to improve survival.

This is of particular importance when dealing with high-risk patients undergoing cardiovascular surgical procedures, in which CO, DO _2_, and perfusion pressure as targets of a goal-directed therapy play a fundamental role in preventing cardiorenal syndrome that may develop and worsen the outcome. This complex disorder is characterized by LCOS secondary to heart failure, acute or chronic kidney dysfunction, and progressive organ involvement, leading to multi-organ failure.

Early signs of LCOS should be promptly recognized or preventively treated, optimizing heart rate and rhythm, improving bi-ventricular contractility, minimizing oxygen demand, and increasing oxygen delivery by using fluids, inotropes, or advanced extracorporeal assist devices (aortic balloon pump, extracorporeal membrane oxygenator, and left ventricular assist device). A recent meta-analysis by Zangrillo *et al.* ^51^ confirmed the beneficial effects on 30-day survival of preoperative intra-aortic balloon pump in high-risk patients undergoing elective CABG.

In case of vasodilatory shock and sepsis, the use of vasopressors such as norepinephrine is mandatory to counteract renal hypoperfusion ^52^. Interestingly, it was recently demonstrated for the first time that, according to published randomized evidence, inotropes and vasoconstrictors do not increase mortality in the perioperative period or in critically ill patients and are probably beneficial in some settings ^53^.

Therefore, inotropes and vasoconstrictors together with fluid and transfusion management are of central importance to prevent kidney injury through early hemodynamic optimization ^54^.

Moreover, fluid overload may have detrimental effects on renal function by ultimately worsening the outcome, especially when considering cardiac surgery patients ^55, 56^. In fact, elevated central venous pressure decreases the driving venous return pressure, possibly leading to interstitial space congestion, abdominal compartment syndrome, and renal congestion ^57^. The last of these results in an increased renal intratubular pressure and a reduced glomerular filtration gradient. In critically ill patients, it may be challenging to distinguish whether fluid overload is a cause or the effect of AKI; however, a positive fluid balance is often associated with AKI ^58, 59^.

Regardless of the amount of fluid administered, the type of fluid may also have an impact on the development of AKI. Current recommendations suggest using isotonic crystalloids for initial fluid expansion (in the absence of hemorrhagic shock), avoiding hydroxyethyl starch (HES) perioperatively, with the aim to prevent or treat AKI ^60, 61^. Uncertainty exists on the nephrotoxic risk when older colloids are administered ^62– 64^ instead of iso-oncotic solutions (third-generation colloids). On the other hand, a recent meta-analysis of randomized trials ^65^ showed no evidence of a higher risk associated with third-generation HESs in cardiac surgery but did recommend further investigations in the future.

Among crystalloid solutions, saline is associated with hyperchloremia ^66^ and reduced renal flow ^67^, whereas buffered crystalloid solutions remain the fluid of choice in critically ill patients ^68^, although a recent RCT showed no reduced risk of AKI with buffered crystalloids compared with saline ^69^. Other RCTs with adequate statistical power and assessing high-risk population are needed for more conclusive results.

## Other treatments

Although optimization of CO and reduction in CPB time are the key factors to reduce AKI incidence in cardiovascular surgery ^70^, several other supportive therapies have been proposed ( Table 2).

**Table 2.** Perioperative renal protection, strategies to be considered in cardiovascular surgery.

*Melius abundare quam* deficere*	Standard patients	Patients at high risk of acute kidney injury
Perioperative hemodynamic optimization	Optimize DO _2_ through Ht, CO, HR, and paO _2_ No specific device or technique is recommended	Optimize DO _2_ through Ht, CO, HR, and paO _2_ with any available device, technique, or protocol
Inotropes and vasoconstrictors	Optimize perfusion pressure	Optimize perfusion pressure Choose a “patient-tailored MAP” (according to the basal MAP)
Fluid management	Avoid routine fluid overload	Target therapy with a GDT protocol
Liberal transfusion therapy	Consider liberal (Hb 9) instead of a restrictive (Hb 7) transfusion strategy	Use liberal (Hb 9) instead of a restrictive (Hb 7) transfusion strategy
Renal replacement therapy	Use it in the early phase of acute kidney injury	Consider it even as a preventive strategy
Volatile	Consider routinely using them in your anesthesia plan of cardiovascular patients	Use them in CABG surgery
Remote ischemic preconditioning	Consider routinely using it in cardiovascular patients if you can avoid propofol during the procedure	Use it if you can avoid propofol
Intra-aortic balloon pump	No	Use it in high-risk CABG patients or in cardiovascular procedures with complicated course
Epidural	Weigh risks and benefits according to your expertise and patients’ preferences	Weigh risks and benefits according to your expertise and patients’ preferences
Levosimendan	No	Use it Avoid bolus Consider vasoconstrictors
Statin	Consider routinely reintroducing them immediately after surgery	Consider starting them before surgery

*Too much is better than not enough. CABG, coronary artery bypass grafting; CO, cardiac output; DO _2_, oxygen delivery; GDT, goal-directed therapy; Hb, hemoglobin; HR, heart rate; Ht, hematocrit; MAP, mean arterial pressure; paO _2_, partial pressure of oxygen in arterial blood.

***Fenoldopam*** has a selective vasodilatory effect on renal circulation and therefore is associated with an increased blood flow. Unfortunately, a recent large RCT demonstrated that fenoldopam infusion does not prevent worsening of AKI after cardiac surgery and is not associated with a reduced need for RRT ^71^. A potential explanation is the underlying multifactorial nature of AKI; fenoldopam may theoretically be an effective treatment in the case of hypoperfusion AKI but not with ischemic insults. As previously mentioned, fenoldopam has also been found to be ineffective in preventing CI-AKI in patients with CKD ^72^.

***Diuretics*** are the most commonly used drugs in critically ill patients for fluid overload management; however, they have shown no effect in AKI prevention and treatment (level of evidence 1B) ^7^ and might be detrimental ^73, 74^. When AKI occurs, RRT represents the main treatment, although optimal timing and dose are still matters of debate ^75, 76^.

***Glucose management*** may prevent the occurrence of AKI. Some studies have reported a benefit whenever glycemic levels were strictly controlled ^77^. However, further RCTs are needed to confirm the involvement of hyperglycemia in the development of AKI and on survival following cardiovascular surgery, and differences might exist between diabetic and non-diabetic patients ^78^.

***Statins***, in this context, play an important role as agents that exert antioxidant, antithrombotic, and anti-inflammatory effects. Indeed, according to an RCT ^79^ and a recent meta-analysis ^80^, statins seem to reduce mortality in patients undergoing cardiovascular surgery.

Curiel-Balsera *et al.* ^81^, on the other hand, in a prospective cohort study analyzing 7276 patients undergoing cardiac surgery, reported no benefit of statin administration in prevention of AKI. These results are in agreement with a previous study performed by Brunelli *et al.* ^82^, in which the effect of statins on AKI was less prominent in the cardiac surgery population (high-risk patients) with respect to a general surgery group. Once again, these findings may be explained by the fact that, in this specific context, AKI is dependent on multiple factors, and simply correcting one single component within a multifactorial picture does not exert sufficient effects to impact the renal outcome.

***The hemoadsorption device***, a relatively new technique used as a filter able to eliminate cytokines and molecules producing cytokines, has potential anti-inflammatory properties. Mostly investigated in animal models of septic shock ^83^ and patients with sepsis-related organ failure ^84^, the hemoadsorption device seems to drastically reduce tumor necrosis factor, IL-6, IL-10, and procalcitonin other than free hemoglobin, myoglobin, and bilirubin. Its use is associated with an improvement in hemodynamic, renal, and liver function and a better outcome in a case report ^85^. In cardiac surgery, retrospective observational studies showed the reliability and safety of the hemoadsorption device in reducing postoperative systemic inflammatory response syndrome ^86, 87^. Therefore, the hemoadsorption device represents a reasonable approach to prevent or improve anti-inflammatory-related renal injury after CPB, although RCTs in cardiac surgery patients are required.

***Aspirin***, a commonly used antiplatelet, has been suggested to be protective for postoperative renal injury. However, the risks associated with this drug, such as perioperative bleeding, may be deleterious for high-risk patient instability and may directly or indirectly provoke AKI ^88^.

## Future perspectives

***RRT before kidney injury*** has been proposed as a prophylactic intervention in patients at particularly high risk of developing kidney dysfunction ^89^. Unfortunately, data supporting a preventive use of RRT in high-risk patients are insufficient ^90^, although an early start of RRT may be beneficial to outcomes in patients with AKI ^91^. The rationale of both early and prophylactic RRT is to restore homeostasis and support residual kidney function as soon as possible within different settings, such as fluid overload, immense pro- and anti-inflammatory response, and nephrotoxic agent-induced nephropathy. Future trials in cardiovascular surgery are warranted to support these data.

***Remote ischemic preconditioning*** is a promising, intriguing, and economic method to reduce postoperative AKI in cardiovascular surgery ^92^. This strategy has additive effects when volatile agents are used because the latter can reduce mortality through cardiac protection and improve renal outcomes through a cardiorenal mechanism ^93^. The beneficial organ-protective effects of remote ischemic preconditioning vanish when intravenous propofol is used ^94^, and this may interfere with organ protection ^95^.

***Levosimendan***, a drug known as a calcium sensitizer with inotropic properties, has recently shown a promising beneficial effect on renal function and a reduction in the need for RRT ^96^ in critically ill patients, including cardiovascular surgery patients. This molecule has beneficial effects on ventricular contraction (inotropic effect) and relaxation (improvement of diastolic function) without increasing myocardial oxygen demand and also has anti-inflammatory qualities ^97^. All these properties, together with its action on venodilation and depression of central venous pressure, may play an important role in reducing renal congestion and increasing renal perfusion pressure. Further well-powered RCTs aiming to validate renal protection by levosimendan are warranted.

***Epidural anesthesia*** has been considered an effective anesthetic strategy, combined with general anesthesia, in improving perioperative outcomes, such as mortality, mechanical ventilation, and myocardial infarction and consequently related organ failure ^98^. Recently, Landoni *et al.*, in an updated meta-analysis, showed a mortality reduction in patients undergoing cardiac surgery with an epidural catheter ^99^ and estimated a risk of epidural hematoma of 1:3552. However, we should always weigh the risk of the procedure, which requires adequate skills and specific care of anticoagulation administration. Therefore, future well-powered RCTs are needed to validate the efficacy of epidural anesthesia.

***Endothelin A-receptor antagonist (ET [A]-RA)***, assessed in animal models ^100^, is a promising therapy for the prevention of post-cardiac surgery AKI. Indeed, the administration of ET (A)-RA during CPB has been associated with a reduction in endothelin-1 (ET-1) and in the numbers of inflammatory cells and an improvement in creatinine clearance. The rationale is that after CPB there is evidence of endothelial dysfunction caused by a reduction in endothelial nitric oxide synthase expression in the vascular endothelium and glomeruli and an increase in ET-1 expression in the tubular epithelium ^100^. ET-1 itself induces renal vasoconstriction that could potentially have a critical pathogenic role in the development and progression of AKI ^101^.

This setting determines loss of endothelial integrity and an oxidative cascade, so that the potential reverse of this insult could be beneficial. Unfortunately, only few animal studies ^102, 103^ support the possible therapeutic role of ET (A)-RA, and further trials on humans are urgently warranted.

## Conclusions

Cardiovascular surgery-related AKI is a common and often dreadful event, frequently associated with higher morbidity and mortality. A number of preventive interventions are currently available, but only a restricted number of them are supported by a reasonable amount of evidence and are therefore recommended. The most feasible measures are to carefully analyze all the preoperative risk factors, in order to optimize medical therapy and management, and to prevent or promptly treat AKI. Timely intervention is of paramount importance because, once nephropathy is established, no available efficacious treatment exists, and this leads to an exponentially increased mortality.

Hence, regardless of the controversies behind the treatment and management of AKI, we strongly suggest a perioperative optimization of renal function, peripheral perfusion, and DO _2_ and an attentive avoidance of nephrotoxic drugs and fluid overload. This is of particular importance in a cardiovascular surgical setting, where patients are exposed to a major risk of developing AKI.

Further fields of research should focus on the identification of underlying risk factors or possible high-risk genetic traits, the investigation of novel and early biomarkers capable of recognizing patients with higher degrees of renal injury, and the development of new pharmacological or mechanical support measures aimed at the reduction of AKI occurrence.
